# Stereotactic radiosurgery for the treatment of brainstem metastases: a multicenter retrospective study

**DOI:** 10.1186/s13014-022-02111-5

**Published:** 2022-08-09

**Authors:** Luca Nicosia, Piera Navarria, Valentina Pinzi, Martina Giraffa, Ivana Russo, Paolo Tini, Niccolò Giaj-Levra, Filippo Alongi, Giuseppe Minniti

**Affiliations:** 1 Advanced Radiation Oncology Department, Cancer Care Center, IRCCS Sacro Cuore Don Calabria Hospital, Negrar di Valpolicella, Italy; 2 Radiotherapy and Radiosurgery Department, Humanitas Clinical and Research Hospital-IRCCS, Rozzano, MI Italy; 3 Department of Neurosurgery, Fondazione IRCCS Istituto Neurologico C Besta, Via Celoria 11, 20133 Milan, Italy; 4 UPMC Hillman Cancer Center, San Pietro Hospital FBF, Rome, Italy; 5 UPMC Hillman Cancer Center, Villa Maria, Mirabella Eclano, AV Italy; 6 Department of Medicine, Surgery and Neurosciences, University of Siena, Policlinico Le Scotte, 53100 Siena, Italy; 7 University of Brescia, Brescia, Italy; 8 IRCCS Neuromed, 86077 Pozzilli, IS Italy

**Keywords:** Brainstem metastases, SRS, stereotactic radiosurgery, SRT, stereotactic radiotherapy, Brain metastases, Linac

## Abstract

**Background:**

Brainstem metastases (BSM) are associated with a poor prognosis and their management represents a therapeutic challenge. BSM are often inoperable and, in absence of randomized trials, the optimal radiation treatment of BSM remains to be defined. We evaluated the efficacy and toxicity of linear accelerator (linac)-based stereotactic radiosurgery (SRS) and hypofractionated steretotactic radiotherapy (HSRT) in the treatment of BSM in a series of patients treated in different clinical centers.

**Methods:**

We conducted a multicentric retrospective study of patients affected by 1–2 BSM from different histologies who underwent SRS/HSRT. Freedom from local progression (FLP), cancer-specific survival (CSS), overall survival (OS), and treatment-related toxicity were evaluated. In addition, predictors of treatment response and survivals were evaluated.

**Results:**

Between 2008 and 2021, 105 consecutive patients with 111 BMS who received SRS or HSRT for 1–2 BSM were evaluated. Median follow-up time was 10 months (range 3–130). One-year FLP rate was 90.4%. At the univariate analysis, tumor volume ≤ 0.4 cc, and concurrent targeted therapy were associated with longer FLP, with combined treatment that remained a significant independent predictor [0.058, HR 0.139 (95% CI 0.0182–1.064]. Median OS and CSS were 11 months and 14.6 months, respectively. At multivariate analysis, concurrent targeted therapy administration was significantly associated with longer OS [HR 0.514 (95%CI 0.302–0.875); p = 0.01]. Neurological death occurred in 30.4% of patients, although this was due to local progression in only 3 (2.8%) patients.

**Conclusion:**

Linac-based SRS/HSRT offers excellent local control to patients with BSM, with low treatment-related toxicity and no apparent detrimental effects on OS. When treated with ablative intent, BSM are an uncommon cause of neurological death. The present results indicates that patients with BSM should not be excluded a priori from clinical trials.

## Introduction

Stereotactic radiosurgery (SRS) given in a single fraction using doses of 18–24 Gy is the current standard treatment for patients with a limited number of brain metastases (1–4), and its use has progressively replaced the use of whole-brain radiotherapy (WBRT) [1–3]; however, patients with brainstem metastases (BSM) [4] are often excluded from prospective trials of SRS because the fear of excessive toxicity caused by exposure of the brainstem to high doses of radiation and its potential negative impact on survival. Therefore, there is a lack of evidence-based recommendations on the optimal radiation treatment of BSM in terms of techniques and dose-fractionation, and the management of these patients in clinical practice remains controversial.

The reluctance to use SRS as treatment of BSM is derived, at least in part, from historical studies demonstrating a maximum tolerated radiation dose for the brainstem of 12–12.5 Gy given as a single fraction [5, 6]. SRS for BMS was described for the first time in 1993 [7]. Since then, several authors reported their retrospective experience, usually small and monoinstitutional [8–18]. These studies did not show an increased risk of toxicity but provided inhomogeneous indication regarding dose prescription and clinical results, also due to heterogeneity in patients’ selection [11, 18–21]. Moreover, the majority of these patients were treated with gamma-knife SRS, with few data available for frameless linear accelerator (linac)-based SRS. A recent systematic review and meta-analysis of those retrospective studies demonstrated the efficacy of SRS in the treatment of BSM; however, only a few predictive parameters of treatment response were identified [22]. Therefore, it is hard to date to infer specific recommendations on dose prescription, as well as the identification of predictive factors of outcome because of the limited available data.

This multicentric retrospective study aims to evaluate the efficacy and safety of frameless linac-based SRS or hypofractionated stereotactic radiotherapy (HSRT) to BSM and to identify factors predictive of tumor control and survival.

## Patient and methods

The present multi-institutional study was conducted on a retrospective cohort of patients with1-2 BSM who received SRS or HSRT between April 2008 and July 2021. Data were anonymously collected in an internal review board (IRB)-approved database. Inclusion criteria were: (a) age > 18 years; (b) diagnosis of BSM confirmed by contrast-enhanced MRI acquired no more than 4 weeks before radiation treatment; (c) Eastern Cooperative Oncology Group (ECOG) performance status ≤ 2. Patients treated with concomitant systemic therapy were included in the study. SRS could have been administered concomitantly to systemic therapy (within one months from the last administration). The study was conducted in accordance with the principles of the Declaration of Helsinki and was approved by the IRB of participating Institutions.

### Treatment characteristics

Patients underwent a CT simulation without contrast media (1-mm slice thickness) for radiation therapy planning with a thermoplastic mask. A co-registration of volumetric CT and the T1 sequences of the MRI (3-dimensional spoiled gradient series with 1-mm slice thickness) no older than 2 weeks was used to define organs at risk (OARs) and target volumes. Gross tumor volume (GTV) encompassed the macroscopic contrast-enhancing lesion on T1-MRI and was assumed equal to the clinical target volume (CTV). The planning target volume (PTV) was obtained from the GTV plus an isotropic margin of 0–2 mm. We performed a risk-adapted dose prescription. SRS with a dose of 16–18 Gy was generally administered for lesions ≤ 10 mm, while HSRT with total doses of 14–32 Gy given in 2–5 fractions was reserved for lesions > 10 mm. The dose was usually prescribed at 80% isodose line and dose optimization was performed to cover 95% of PTV with the prescription dose. Treatment was administered with a linac using either volumetric-modulated arc therapy (VMAT), or multiple dynamic arcs (DCAT) or fixed beams.

### Follow-up

Physical examination, toxicity assessment, and radiological response with MRI were performed after 45–60 days following the first treatment. Subsequently, follow-up was performed every 3 months for the first 2 years and every 4–6 months for the next 3 years. Tumor response was evaluated using the Response Assessment in Neuro-Oncology (RANO) criteria [23]. MR response assessment was based on contrast enhanced T1-w and fluid attenuated inversion recovery (FLAIR) sequences. Toxicity was assessed during and after radiotherapy according to the Common Terminology Criteria for Adverse Events (CTCAE) v5.0. Acute toxicity was defined as an adverse event occurring within 90 days from the beginning of treatment, whereas late toxicity after 90 days.

### Study end-points and statistics

The primary end-point was freedom from local progression (FLP). Secondary endpoints were overall survival (OS), cancer-specific survival (CSS), neurological death (ND), and toxicity. Survivals were defined as follows: FLP as the time between SRS administration and the occurrence of in-field or marginal regrowth of the disease; OS was defined as the time to death or last follow-up; CSS as the time to death due to tumor progression or last follow-up, and ND as the death due to brain disease progression.

The univariate analysis was performed with the Kaplan–Meier method. The log-rank test was applied to determine differences between the corresponding curves. The following covariates were evaluated for survival end-points: sex, age, lesion volume (cc), PTV margin, BSM site (midbrain, pons, medulla oblongata) biological effective dose (BED), previous WBRT, primary tumor histology, concomitant therapy (target therapy, immunotherapy, chemotherapy), fractionation. Univariate and multivariate analyses were performed by the Cox proportional hazards model. Clinically relevant variables with a p < 0.1 at univariate analysis were included in the multivariate analysis. The threshold of tumor volume related to SRS response and/or survival was determined with the ROC curve method, calculating the highest product of (sensitivity*specificity). BED calculations for different radiation schedules was determined by linear-quadratic model according to an α/β ratio of 10 Gy for tumors. Statistical analysis was performed using the SPSS statistical software package version 26.0 (SPSS Inc, Chicago, IL). A p-value ≤ 0.05 indicated a significant association.

## Results

### Patients’ characteristics

The patient population was represented by 105 patients with 111 BSM. Patient and treatment characteristics are shown in Table 1. Main primary histology were non-small cell lung cancer (NSCLC, 46.6%), breast cancer (22%), renal cell carcinoma (10.5%), and melanoma (9.5%). Concomitant systemic therapy was administered in 76 patients, and included chemotherapy (26.2%), target therapy (25%), immunotherapy (13.5%), and hormone therapy (2.8%). More specifically, targeted therapies consisted of anti-HER2 agent (n = 12), ALK inhibitor (n = 7), tyrosin-kinase inhibitor (n = 6), and VRAF inhibitor (n = 3). The median follow-up was 11 months (range 3–130). RT treatment consisted of single-fraction SRS for 58 (52.3%) lesions and HSRT (2–5 fractions) for 53 lesions (47.7%). The median administered BED10 was 35.7 Gy (range 23.8–60). The GTV volume threshold for survival analysis was 0.4 cc (AUC 0.69, 95%CI 0.61–0.74; p = 0.02). Median BSM volume was 0.4 cc (range 0.02–23.6).

**Table 1:** Patients’ and lesions’ characteristics (n = 105)

*Age, median (range)*	58 (36–85)
*Primary tumor*	
Lung	49 (46.6%)
Breast	23 (22%)
Kidney	11 (10.5%)
Melanoma	10 (9.5%)
Gastrointestinal	5 (4.8%)
Gynecological	5 (4.8%)
Head & neck	1 (0.9%)
Bladder	1 (0.9%)
*Concomitant systemic therapy*	
Chemotherapy	29 (26.2%)
Targeted therapy	28 (25%)
Immunotherapy	15 (13.5%)
Hormone therapy	3 (2.8%)
None	29 (26.2%)
Unknown	7 (6.3%)
*Previous WBRT*	
Yes	15 (14%)
No	90 (86%)
*Brainstem site (n* = *111)*	
Midbrain	33 (30%)
Pons	63 (57%)
Medulla oblongata	15 (13%)
*Fraction number (n* = *111)*	
1	58 (52.3%)
2	1 (0.9%)
3	38 (34%)
4	2 (1.8%)
5	12 (11%)
*Median total dose SRS (Gy) (range)*	18 (12–20)
*Median total dose HSRT (Gy) (range)*	20 (14–32)
*Median BED (Gy10) (range)*	35.7 (23.8–60)
*Median/mean GTV volume SRS (cc) (n* = *58)*	0.4/1.3 (0.02–2.8)
*Median/mean GTV volume HSRT (cc) (n* = *53)*	0.4/1.5 (0.08–23.6)

WBRT: whole-brain radiotherapy; SRS: stereotactic radiosurgery; HSRT: hypofractionated stereotactic radiotherapy, GTV: gross tumor volume

### Freedom from local progression and toxicity

**Fig. 1:**
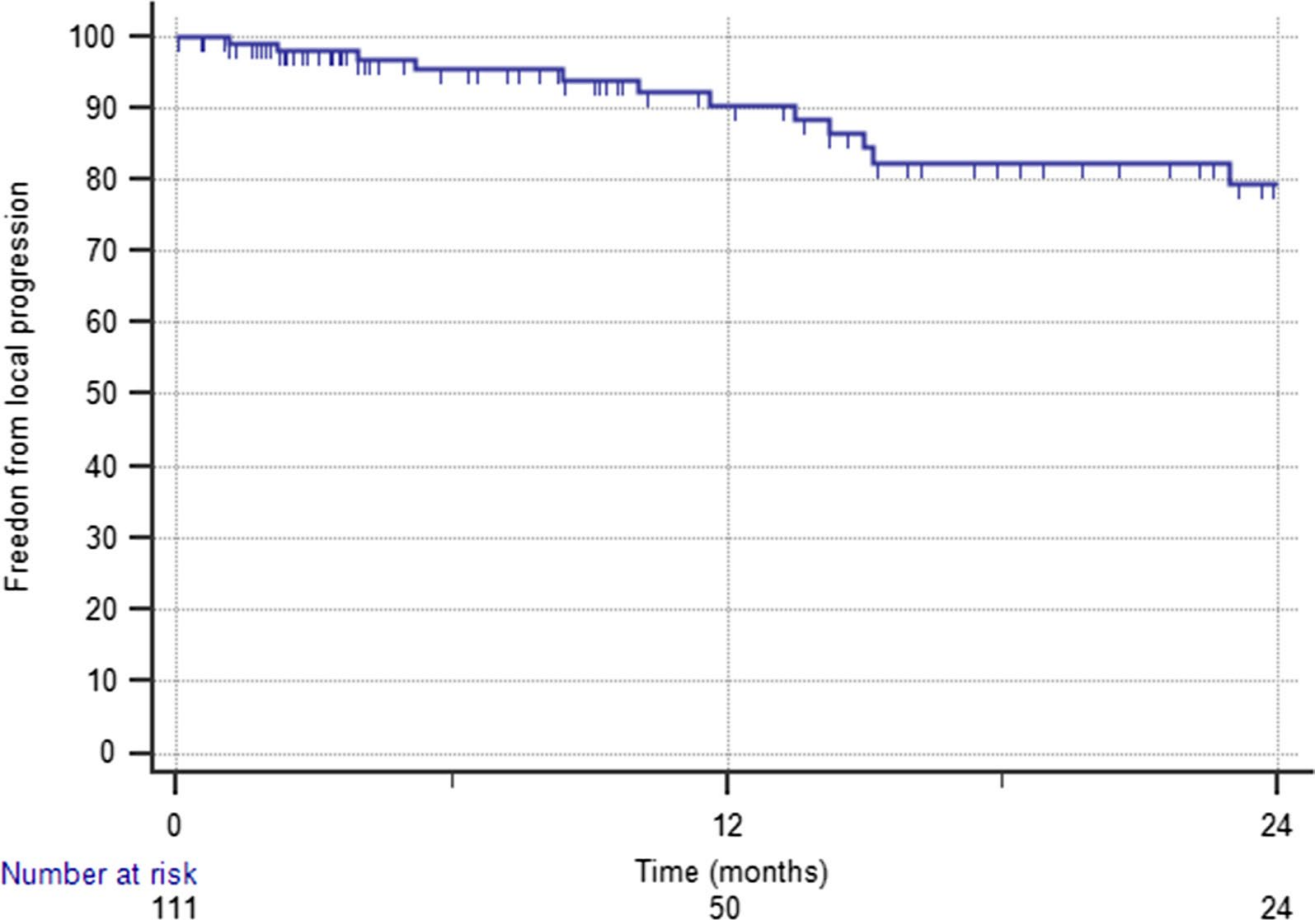
Kaplan–Meier curve showing freedom from local progression in the overall population

**Fig. 2:**
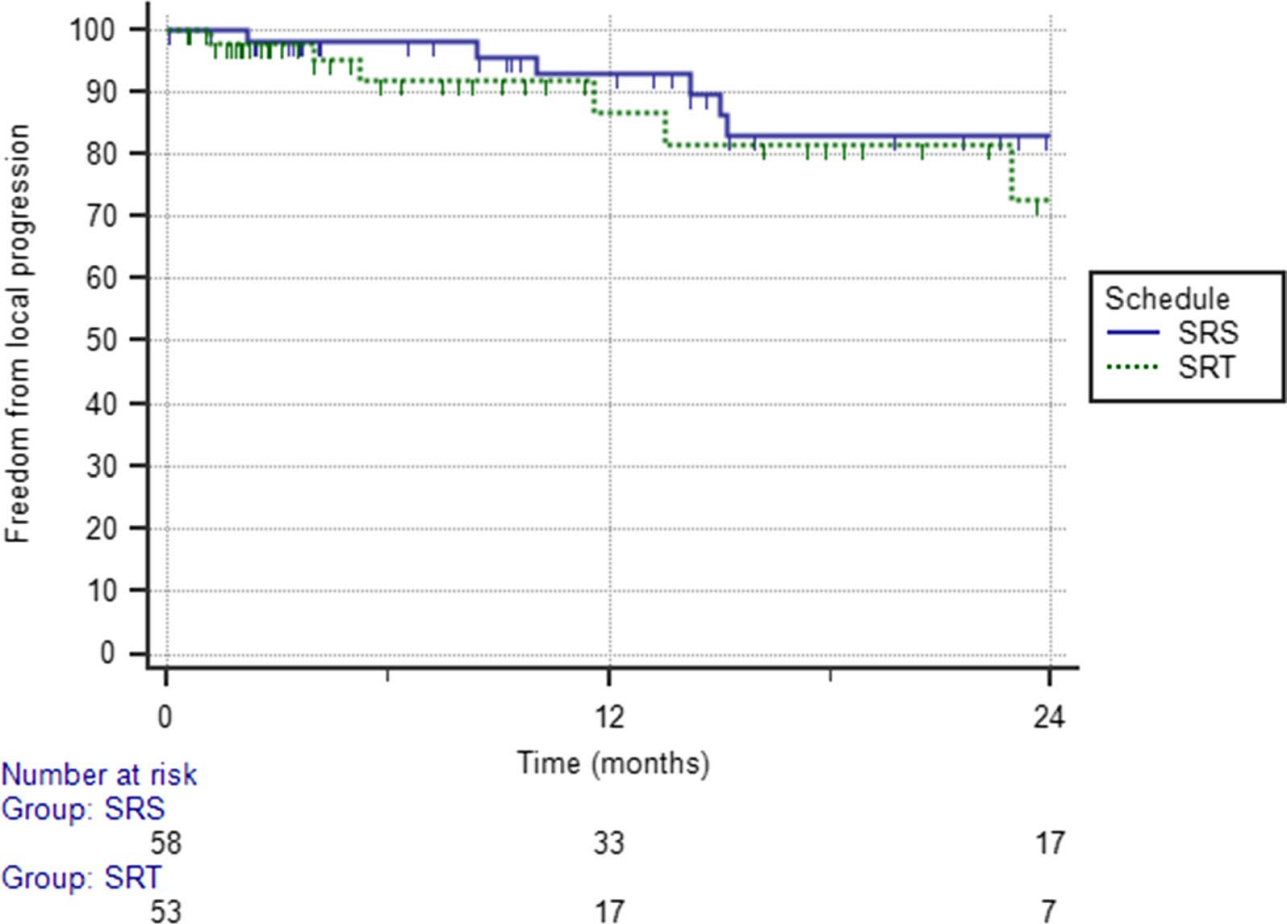
Kaplan–Meier curve showing freedom from local progression stratified according to fractionation (stereotactic radiosurgery (SRS) versus hypofractionated stereotactic radiotherapy (HSRT))

With a median time of 11 months, the 1-year FLP was 90.4%, with 13 lesions that progressed at a median time of 10 months (Fig. 1). Results of univariate and multivariate analysis are shown in Table 2. GTV volume ≤ 0.4 cc, and concurrent targeted therapy were predictive of better FLP. In particular, 1-year FLP rates were 98.1% for a GTV volume ≤ 0.4 cc and 82.9% for a GTV volume > 0.4 cc (p = 0.041). 1-year FLP was 96.5% following combined treatment and 86% after SRS/HSRT alone (p = 0.03), with no difference between SRS and HSRT (Fig. 2; p = 0.51). At the multivariate analysis, pons location was an independent factor of improved FLP [HR 0.202 (95%CI 0.048–0.845); p = 0.02], while concurrent targeted therapy was of borderline significance [0.058, HR 0.139 (95% CI 0.0182–1.064]. No severe acute toxicity occurred. A case of grade 2 pseudoprogression was recorded in one patient 3 months after the treatment, and grade 2 headache in 3 patients, who were successfully treated with steroids. For these patients, perfusion MRI changes were suggestive of symptomatic radiation necrosis. No grade 3 or higher acute toxicity occurred.

**Table 2:** Uni- and multivariate analysis

	Univariate analysis	Multivariate analysis
	Freedom from local progression	
BED 35.7 Gy10	0.13	HR 0.455 (95%CI 0.140–1.479); p = 0.19
GTV volume ≤ 0.4	*0.041*	HR 3.77 (95%CI 0.945–15.061); p = 0.06
PTV margin	0.59	–
BM site (base = midbrain)	0.09	*Pons: HR 0.202 (95%CI 0.048–0.845); p* = *0.02* M. Oblongata: HR 0.627 (95%CI 0.150–2.619); p = 0.52
Primary histology	0.74	–
Previous WBRT	0.85	–
Concurrent targeted therapy	*0.03*	HR 0.169 (95%CI 0.021–1.348); p = 0.09
Single- versus multifraction	0.51	–
	Overall survival	
BED 35.7 Gy10	0.06	HR 0.887 (95%CI 0.534–1.473); p = 0.64
GTV volume ≤ 0.4	0.45	–
PTV margin	0.17	–
Primary histology	0.17	
BM site	0.16	–
Previous WBRT	*0.01*	HR 1.59 (95%CI 0.827–3.080); p = 0.16
Concurrent targeted therapy	*0.01*	*HR 0.466 (95%CI 0.270–0.804); p* = *0.006*
Single-versus multifraction	*0.03*	HR 1.61 (95%CI 0.972–2.696); p = 0.06
	Cancer-specific survival	
BED 35.7 Gy10	0.26	–
GTV volume ≤ 0.4	0.33	–
PTV margin	0.23	–
BM site (base = midbrain)	0.06	Pons: HR 0.587 (95%CI 0.338–1.019); p = 0.06 M. oblongata: HR 1.026 (95%CI 0.505–2.082); p = 0.94
Primary histology	0.23	
Previous WBRT	*0.03*	*HR 2.055 (95%CI 1.023–4.128); p* = *0.04*
Concurrent targeted therapy	*0.03*	*HR 0.573 (95%CI 0.331–0.992); p* = *0.04*
Single- versus multifraction	0.11	–
	Neurological Death	
BED 35.7 Gy10	0.75	–
GTV volume ≤ 0.4	0.75	–
PTV margin	0.54	–
Primary histology	0.23	
BM site (base = midbrain)	0.15	Pons: HR 0.431 (95%CI 0.179–1.037); p = 0.06 M. Oblongata: HR 0.631 (95%CI 0.172–2.308); p = 0.48
Previous WBRT	*0.00*	*HR 3.381 (95%CI 1.287–8.879); p* = *0.01*
Concurrent targeted therapy	0.13	0.530 (95%CI 0.197–1.427); p = 0.21
Single- versus multifraction	0.74	–

*BED*: biological effective dose; *HR*: hazard ratio; *CI*: confidence interval; *GTV*: gross tumor volume; *PTV*: planning target volume; *BM*: brainstem; *WBRT*: whole-brain radiotherapy

Italics values indicate a significant correlation

### Overall Survival and prognostic factors

**Fig. 3:**
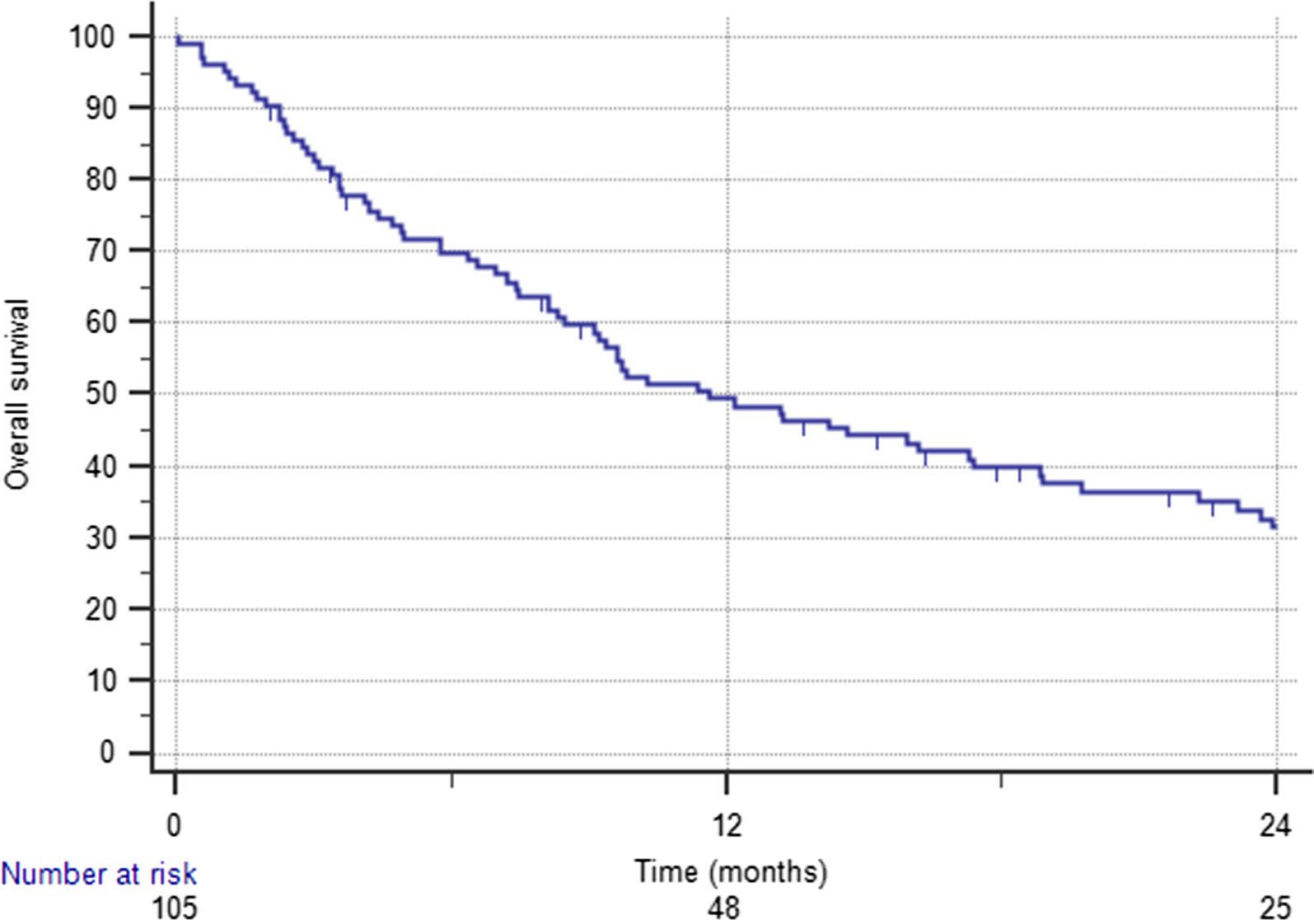
Kaplan–Meier curve showing overall survival

The median OS time was 11 months (range 9–17.4) and 1-year OS rate was 49.5% (Fig. 3). The median CSS time was 14.6 months (range 10–22.3). The 1-year CSS rate was 55.6%. In total, 32 patients (30.4%) had ND. The 1-year ND rate was 77.5%. At the last follow-up, 84 patients (80%) died. Forty-eight (45.7%) died of systemic progression and 32 (30.4%) died of intracranial progression; however, this was due to local BSM progression only in 3 patients. In four (3.8%) patients, death resulted from noncancer causes.

At the univariate analysis, previous WBRT was associated with worse 1-year OS (30.5% versus 52.6%, p = 0.01), CSS (35.6% versus 58.6%, p = 0.03), and ND rates (57.9% versus 80.6%, p = 0.001). Concurrent target therapy was a predictor of better OS (67.5% versus 45.8%, p = 0.01) and CSS (70.2% versus 48.2%, p = 0.03) rates at 1 year. Similarly, SRS was associated with improved OS (p = 0.03). At the multivariate analysis, concurrent targeted therapy remained significantly associated with improved OS [HR 0.514 (95%CI 0.302–0.875); p = 0.01] and CSS [HR 0.573 (95%CI 0.331–0.992); p = 0.04]. Previous WBRT was an independent factor of lower CSS [HR 2.055 (95%CI 1.023–4.128); p = 0.04] and ND [HR 3.381 (95%CI 1.287–8.879); p = 0.01].

## Discussion

Brainstem metastases in cancer patients are usually associated with poor survival and quality of life. The presence of vital structures justifies the rapid onset of symptoms even when the lesion dimension is small. For these inoperable lesions, radiotherapy has historically been the most widely used therapeutic option. The use of SRS was generally limited by the fear of side effects; however, an accumulating body of literature in the last two decades is demonstrating its efficacy in patients with BSM.

Yet, the largest published series is a multi-institutional analysis by Trifiletti et al. [24] including 547 patients with BMS who were treated with Gamma Knife SRS. The 1-year local control and FLP rates were 81.8% and 90.4%, respectively, using a median marginal dose of 16 Gy. In the univariate analysis, a marginal dose < 16 Gy was associated with worse local control; however, the correlation was not confirmed in the multivariate analysis. These results compare with those reported in series of SRS for cerebral and cerebellar metastases, reporting 1-year local control rates of 86.7% to 95% [2, 25].

Differently from other studies, in the current series a significant proportion of patients received HSRT, typically 14–32 Gy given in 2–5 fractions. The 1-year FLP rates were similar, 92.9% and 86.8% after SRS and HSRT, respectively, although fractionated SRS was used more often for larger lesions. Based on our results, both approaches offer similar excellent long-term tumor control, at least for a BED 10 > 35 Gy, with low treatment-related toxicity.

BSM have been generally linked to poor survival due to the rapid onset of symptoms, indicating local tumor progression as a frequent cause of cancer death. However, the reported survival in our and other published SRS series might suggest a different scenario. The median cancer-specific survival observed in our study was 14.6 months, being similar to those reported by others [21]. In the study of Trifiletti et al. [24], median OS time and 1-year survival rates were 5.6 months and 32.7%, respectively. Interestingly, death rate for BSM progression was 0.7%, and ND was 16%. In another large Gamma Knife series by Kawabe et al. [12], the death rate for patients with BSM progression was 2.3% and ND rate was 10.9%. In our series, we observed a similar death rate of 2.8%, confirming that SRS is an effective treatment associated with a low mortality rate due to BSM progression.

A point for discussion comes from the role of WBRT in the management of BM. For patients with a limited number of brain metastases, SRS has become the recommended treatment over WBRT. In addition, recent evidence demonstrated that focal irradiation may have a role also in patients with further intracranial progression after a first course of SRS [26, 27]. In the current study, patients who received a previous WBRT had worse CSS, suggesting that SRS should be always considered in all patients with limited brain disease, even in presence of BSM.

Our study has several limitations due to its retrospective nature and selection biases; patients were treated with different radiation schedules, and a subgroup received targeted therapy which might have influenced clinical outcomes. Nevertheless, this is the largest series of linac-based SRS for BSM, and our data on local control and the cause of death strongly support the use of SRS/HSRT in these patients confirming the excellent results reported in previous smaller mono-institutional series (see Table 3) [10, 11, 21, 28–30]. Of note, the rate of local control observed with frameless linac-based SRS/HSRT is in line with other SRS techniques, and our study adds evidence to the role of frameless linac-based SRS in the treatment of BSM.

**Table 3:** Literature review on linac-based SRS on BSM

Study	Population	Metastases	Median prescription dose (range)	Local control	Survival (median)	Neurological death rate	Death by local progression rate	Toxicity
Samblàs et al. [28]	28	30	Median 11.1 Gy (5–20)	n/a	16.8 months	42.8%	3.5%	n/a
Hatiboglu et al. [10]	60	60	Median 15 Gy (8–18)	1-y: 35%	4 months	7%	2%	Hemiparesis (6.6%) Nausea/vomiting (6.6%) Headache (5%) Cranial nerve deficit (3%) Hemorrage (3%)
Kelly et al. [29]	24	24	Median 13 Gy (8–16)	1-y: 78.6%	5.3 months	12.5%	0%	Ataxia (4%) Acute confusion (4%)
Valery et al. [21]	30	43	Median 13.4 Gy (8.2–15)	1-y: 79%	10 months	42% (8 of 19 assessable cases)	n/a	Headache (13%)
Lin et al. [11]	45	48	Median 14 Gy (10–17)	1-y: 88%	11.6 months	n/a	n/a	Overall: 4.7%
Sugimoto et al. [30]	24	25	24–40 Gy/7–13 fr	96%	9 months	n/a	0%	Nausea (4%)
Present study	105	111	35.7 Gy10 (23.8–60)	1-y: 90.4%	11 months	24.7%	2.8%	Headache (2.8%) Pseudoprogression (1%)

In conclusion, frameless linac-based SRS/HSRT is a safe and effective treatment associated with excellent local control in patients with BSM, similar to those reported for lesions in other regions of the brain, with no detrimental effect on survival. Death due to BSM is in fact a rare event, and the presence of BSM in cancer patients should not be considered an exclusion criterion from clinical trials.

## Data Availability

The datasets used and/or analyzed during the current study are available from the corresponding author on reasonable request.

## Abbreviations

BED: Biological effective dose
BSM: Brainstem metastases
CI: Confidence interval
CSS: Cancer-specific survival
CTCAE: Common Terminology Criteria for Adverse Events
ECOG: Eastern Cooperative Oncology Group
FLP: Freedom from locap orgression
GTV: Gross tumor volume
HR: Hazard ratio
IRB: Internal review board
MRI: Magnetic resonance imaging
ND: Neurological death
OAR: Organ at risk
OS: Overall survival
PTV: Planning target volume
RECIST: Response evaluation criteria in solid tumors
SRS: Stereotactic radiosurgery
HSRT: Hypofractionated stereotactic radiotherapy
VMAT: Volumetric-modulated arc therapy
WBRT: Whole-brain radiotherapy
